# The effect of zoledronic acid on hip geometry in renal transplant recipients: a double-blind placebo-controlled randomized study

**DOI:** 10.1186/s12882-023-03376-y

**Published:** 2023-11-08

**Authors:** Alireza Dabbaghmanesh, Marzieh Bakhshayeshkaram, Sharareh Roshanzamir, Arzhang Naseri, Mohammad Mahdi Dabbaghmanesh, Seyed Taghi Heydari, Pedram Talehzadeh, Mohammad Hossein Dabbaghmanesh, Shahrokh Ezzatzadegan Jahromi

**Affiliations:** 1 Endocrinology and Metabolism Research Center, Shiraz University of Medical Sciences, Shiraz, Iran; 2 Department of Physical Medicine and Rehabilitation, Shiraz University of Medical Sciences, Shiraz, Iran; 3 Health Policy Research Center, Shiraz University of Medical Sciences, Shiraz, Iran; 4 Nephrology-Urology Research Center, Shiraz University of Medical Sciences, Shiraz, Iran

**Keywords:** Bone mineral density, Trabecular bone score, Hip structural analysis, Kidney transplant, Zoledronic acid

## Abstract

**Background:**

In renal transplant patients, bisphosphonates may prevent bone loss, but little is known about their effects on bone microarchitecture and geometrical hip parameters, as the key factors of bone stability. This study aimed to analyze the effect of zoledronic acid on the mentioned parameters in kidney transplant patients.

**Methods:**

In this double-blind, randomized trial, 33 patients were followed for six months after administering either 4mg of zoledronic acid or a placebo. Bone mineral density (BMD) measurement of the spine, hip, radius, and whole body was obtained, and trabecular bone score (TBS) was evaluated using the software. Geometric assessment at the proximal femur was performed by the HSA program.

**Results:**

Eighteen patients in the intervention group and 15 in the control group completed the study. The mean percentages of the changes in the BMD at the lumbar spine and whole body were significantly different between the placebo and intervention groups (-0.23% vs. 4.91% and -2.03% vs. 1.23%) (*P* < 0.05). Zoledronic acid appeared to enhance the subperiosteal diameter, endocortical diameter, and cross-sectional moment of inertia (CSMI) at the narrow neck in comparison with placebo (*P* < 0.05); however, no difference in TBS was observed between both groups (*P* > 0.05).

**Conclusions:**

We concluded that a single administration of zoledronic acid might ameliorate bone loss at the lumbar spine and the whole body and maintain the subperiosteal diameter, endocortical diameter, and CSMI as parameters of bone strength at the narrow neck of the proximal femur after six months in renal-transplant recipients.

**Trial registration:**

This study was registered in IRCT (ID: IRCT20181202041821N1) on 04–05-2019.

## Background

The number of end-stage renal disease (ESRD) patients continues to rise, and kidney transplantation provides the opportunity to replace the failed kidneys. However, kidney transplant patients are at a greater risk of complications [1, 2]. The survival of kidney transplant recipients has been significantly increased by immunosuppressive therapy [3], but these medications are the major causes of osteoporosis, which can result in multiple fractures [4]. Some studies have shown a decrease of 6.8% and 8.8% in lumbar bone density 6 and 18 months after kidney transplantation [5]. The study showed that the fracture rate of kidney transplant recipients was approximately four times that of the general population, with an estimated 22.5% of patients suffering a fracture within the first five years following transplantation [6]. It has been shown that a notable portion of the decrease in bone mineral density (BMD) occurs early after kidney transplantation. This reduction ranges from 4 to 10 percent within six months following the transplant [7]. Although this has been known for decades, to date, an appropriate preventive strategy has yet to be established. Thus, finding suitable treatments to ameliorate these patients' bone conditions is necessary. Bone strength mainly reflects the integration of bone density and quality, including material and structural properties. Although bone biopsy is considered the gold standard used to evaluate bone health in renal bone disease, this procedure is performed exceptionally in daily clinical practice. Bone mineral density (BMD), trabecular bone score (TBS), and hip structural analysis (HSA) variables are representative surrogate markers used to assess the efficacy of osteoporosis therapy [8]. HSA is an important factor in forecasting the occurrence of hip fractures. Thus, not only should bone mineral density be measured clinically, but it is also essential to consider HSA meticulously for the risk of hip fracture [9]. Most studies on the assessment of bone loss after renal transplantation were based on BMD attained by dual-energy X-ray absorptiometry. However, immunosuppressive therapy is associated with lower optimal hip structural geometry [10]. The trabecular bone score is a texture quantity derived from dual-energy X-ray absorptiometry lumbar spine images, providing information independent of bone mineral density. Renal transplant recipients had abnormal bone texture measured by TBS, and a lower lumbar spine and TBS were associated with fractures in kidney transplant recipients [11]. Bisphosphonates improve bone density by decreasing the number of osteoclasts and preventing their activity; it has also been shown to be effective in preventing and treating bone loss in postmenopausal osteoporosis [12]. Nonetheless, it is not clear whether the generalized use of bisphosphonate drugs reverses or prevents bone loss after transplantation [13]. Oral bisphosphonate therapy can cause different gastrointestinal side effects, including nausea, difficulty swallowing, heartburn, irritation of the esophagus, and gastric ulcer. Zoledronic acid is a bisphosphonate, which, when administered through annual intravenous infusion, increases BMD and reduces the incidence of fractures in glucocorticoid-induced osteoporosis; also, it has a potential advantage of increasing the compliance and adherence of patients when it is done annually [14]. It has been demonstrated that the administration of zoledronic acid has a beneficial effect in preventing bone loss in the first six months after kidney transplantation, which was determined by increasing the lumbar spine's BMD and stabilizing the femur's BMD during this period [15]. Despite numerous studies regarding the best treatment options for post-transplant bone loss, controversy continues [16]. To the best of our knowledge, no study has examined the effect of bisphosphonate on hip structural analysis and trabecular bone score for the prevention of post-transplant osteopathy. Thus, we performed a randomized, double-blind, placebo-controlled trial of a potent intravenous bisphosphonate, zoledronic acid, for six months on BMD, trabecular bone score, and bone strength in patients who had undergone kidney transplantation.

## Methods

### Study patients

Forty-two primary kidney-only adult recipients, above 18 years of age, who received a kidney transplant from a living donor and had stable graft function, along with an eGFR of more than 30 ml/min per 1.73 m2 within two weeks of transplantation were enrolled in the study. Patients with a history of prior transplantation, immunosuppression, cancer, rheumatoid arthritis, hypo- or hypercalcemia, adynamic bone disease (PTH levels below 150ng/L), pregnancy, weight over 105 kg, previous parathyroidectomy, treatment with corticosteroids for more than three months before transplantation, treatment with calcitonin and bisphosphonates, and parathyroid level of more than 800 pg/ml were excluded.

### Study design and intervention

**Fig. 1:**
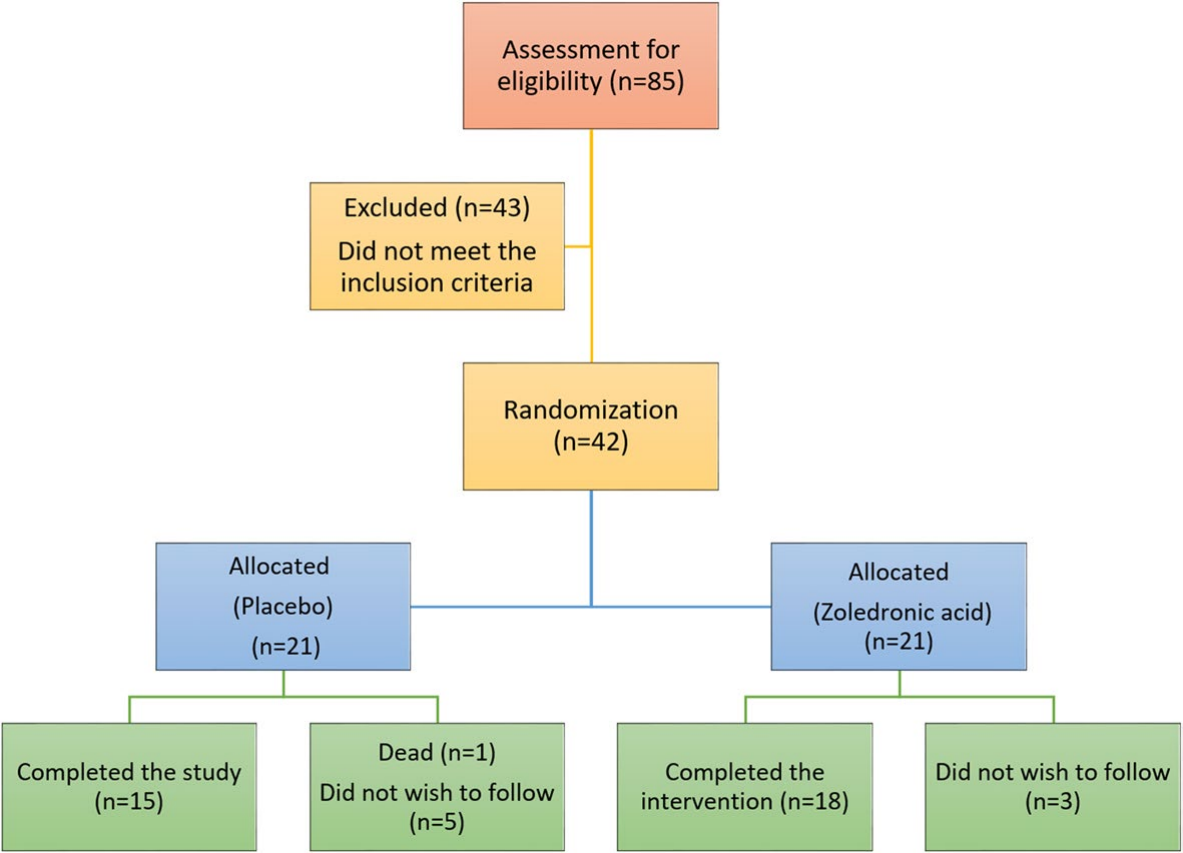
CONSORT flow diagram. Participants' flow throughout the study

This study is a controlled, double-blind 6-month randomized clinical trial. From March 2020 to September 2021, 85 patients who had undergone transplantation for chronic kidney disease in Abu Ali Sina Organ Transplant in Shiraz, Iran, were screened. Forty-two patients who met the inclusion criteria after transplant were randomly allocated with a 1:1 allocation ratio parallelly using random block sizes by Stata statistical software into the intervention and control groups (Fig. 1). The patients were randomly assigned via a computer-generated number system to one of the two groups to receive an intravenous infusion of 4 mg zoledronic acid (Zolena, 4mg/5mL, Ronak Daroo, Iran) within two weeks of transplantation or placebo in 250 mL saline over 15 min. Both groups received the same dose of oral calcium carbonate (1000 mg/d) and vitamin D3 (800 IE/d) supplements, which are typically prescribed for patients undergoing prolonged steroid therapy for different underlying conditions [17]. The clinical staff was aware of which participants were allocated to the intervention group, but outcome assessors and data analysts were blinded to it. All patients underwent maintenance immunosuppressive therapy with triple-agent immunosuppression with tacrolimus, mycophenolate mofetil, and corticosteroids. Methylprednisolone 1 g was intravenously injected on the first day of the operation. Induction was performed with Thymoglobulin 1.5 mg/kg per day, starting during the operation for four days. There were no statistically significant differences between the two groups in tacrolimus levels or cumulative doses of oral corticosteroids during the study period. BMD at the lumbar spine, femoral neck, total hip, whole body, trabecular bone score, and vertebral fracture radiologic assessment was evaluated at baseline (within two weeks after transplantation) and six months after transplantation. All bone densitometry, trabecular bone score, and hip geometry analyses were completed by a certified technician.

There were no significant changes in the trial methods or outcomes after the start of the experiment. We used the Consolidated Standards for Reporting of Trials (CONSORT) statement in order to improve the quality of this randomized controlled trial (RCT) report [18].

### Data collection and measurements

Researcher-made questionnaires were used to collect demographic information, including lifestyle factors, and clinical information. A portable, wall-mounted electronic stadiometer (Seca Model 769; Seca Corp, CA, USA) was used to measure height and weight without shoes. The body mass index (BMI) is calculated by dividing weight (kg) by height squared in meters (kg/m^2^).

As part of the study, tobacco and alcohol habits were asked about, and clinical data such as blood pressure, heart rate, reproductive history (e.g. age of menarche, parity, and age of menopause), and also medical history (e.g. previous and current use of pharmacological therapies, and previous fracture) were collected from participants.

The level of physical activity was evaluated by the International Physical Activity Questionnaire (IPAQ) created by the World Health Organization (WHO). The questionnaire categorized physical activity into three levels: vigorous intensity, moderate intensity, and walking. It also gathered information about the frequency and duration of each activity. The Total METs, a continuous score from the IPAQ scoring protocol, were calculated based on the following formula: (daily minutes of walking per week multiplied by 3.3) plus (daily minutes of moderate-intensity activity per week multiplied by 4.0) plus (daily minutes of vigorous activity per week multiplied by 8.0) [19].

Collection of these data and Measurements of Bone Densitometry, Trabecular Bone Score, and Hip Geometry Analysis was done in the bone densitometry clinic of the endocrinology and metabolism research center of Shiraz University of Medical Sciences.

### Biochemical and hormonal analysis

Blood samples were collected from participants in a clinical laboratory while the subjects were fasted for serum 25(OH) vitamin D, calcium, phosphorus, Parathyroid hormone (PTH), alkaline phosphatase (ALP), and creatinine at baseline (two weeks after transplant) and six months after transplant. Serum levels of 25(OH) vitamin D (reference range [RR]: 20–80 ng/ml) were measured by high-performance liquid chromatography (Young Lee 9100, South Korea). An A25 auto-analyzer (Biosystems SA, Barcelona, Spain) was used to measure calcium (RR: 8.5–10.5 mg/dl), phosphorus (RR; 3.7–5.4 mg/dl), and ALP (RR: 44–147 U/L) levels. Based on sandwich technology, PTH levels (RR: 10–65 ng/L) were checked with ELISA kits by MyBioSource company (USA). eGFR was calculated according to The CKD-EPI equation, expressed as GFR = 141 * min(Scr/κ,1)α * max(Scr/κ, 1)-1.209 * 0.993Age * 1.018 [if female] * 1.159 [if black].

### Measurement of bone densitometry, trabecular bone score, and hip geometry analysis

Areal BMD was determined at the lumbar spine, femoral neck, total hip, and whole body using a Hologic Horizon (Hologic Corp, Bedford, MA, USA) by a qualified technologist according to standard protocols. For vertebral bone mineral density, we measured BMD from L1 to L4. The densitometer was standardized by Phantom before each assessment. The bone mineral density was measured in the lumbar spine, proximal hip, and radius (ultra-distal, mid, one-third, and total). The coefficients of variation for DXA measurements were 0.8%, 1.8%, 0.9%, and 1% for the spine, hip, radius, and total body measurements, respectively. Bone density was stated in grams per centimeter squared. The BMD data were also expressed as T-scores, i.e., SDs below the mean BMD for young adults; also, Z-score was used to compare the bone density to the average values for a person of the same age using the reference ranges provided by the densitometer manufacturer. Osteoporosis and osteopenia were defined as T scores ≤ -2.5 and between -1 and -2.5, respectively [20].

### Trabecular bone score

Trabecular bone score values of the same lumbar vertebrae were calculated based on DXA images using software (TBS iNsight, version 2.1.2.0, Medimaps, Mérignac, France). The software takes the anteroposterior spine raw image(s) from the densitometer (Hologic Corp, Bedford, MA, USA), including the BMD region of interest; therefore, the TBS calculation was conducted over the same region of interest as the BMD measurement. The reproducibility of TBS assessments in several mono-center studies was reported between 1.1% and 1.9% CV [21].

### Hip structural analysis

At three regions of interest (ROIs), narrow neck (NN), inter-trochanter (IT), and femoral shaft (FS), bone geometric indices were assessed using the HSA program included in APEX software (v3.2, Hologic Inc., Waltham, MA, USA).

The narrow neck (NN) region is the narrowest width of the femoral neck, the inter-trochanteric (IT) region is on the bisector of the angle between the axes of the neck and femoral shaft, and the femoral shaft (FS) region is 2 cm distal to the patient's lesser trochanter midpoint.

In all three regions discussed above, the following HSA geometric indices were analyzed: Sub-periosteal diameter (cm); Endo cortical diameter (cm); Cross-sectional area (CSA) excluding soft spaces in the marrow and pores as a reflector of resistance to forces along the long axis (cm2); Cross-sectional moment of inertia (CSMI) which represents resistance to bending forces in a cross Section (cm4); Section modulus (Z) as maximal stress with bending forces index (cm3); Cortical thickness (cm); Buckling ratio (BR) which is the outer radius to wall thickness ratio that is an indicator of the susceptibility to fracture by buckling under compressive load; and the Neck shaft angle that is the angle of the long axes of the femoral shaft and the femoral neck.

The coefficients of variation for NN, IT, and FS regions in our laboratory were < 3%.

### Radiographic assessment

Thoracolumbar radiographs were obtained in all patients to detect vertebral fractures at the time of enrollment and after six months. New vertebral fractures were identified and classified according to the Genant grading system [22]. Fracture assessments were performed by one observer.

### Statistical analysis

The primary endpoints of change in BMD in the lumbar spine were used as the basis for a sample size calculation. According to Coco et al. [23] that showed the mean ± SD changes of -5.81% ± 0.09% in the control group and -0.39% ± 0.05 in the bisphosphonate group in the lumbar spine BMD, eight patients in each group were calculated as a sample size to find similar results with zoledronic acid (α = 0.05 and β = 0.1); however, since there have not been recent studies on the subject and BMD has been found to remain more stable at central sites in post-transplant recipients with current immune suppressive protocols, the sample size was determined based on the findings of a pilot study involving ten patients in a pre-and post-design manner at a = 0.05. It was calculated that 15 patients were required to provide 90% statistical power of the study in each group.

The Kolmogorov–Smirnov test was performed to determine whether data were normally distributed. Mean (standard deviation) or median (interquartile range) and frequency (percentage) were reported for quantitative and qualitative variables, respectively. The continuous variables were compared between groups using independent t-test or Mann–Whitney U test when appropriate, and frequencies were compared using the Chi-square test. *P* values below 0.05 were considered significant in the analysis of the data by SPSS software version 18.

## Result

### Baseline clinical characteristics

Of forty-two patients in this study, thirty-one (73.8%) were men and 11 (26.2%) were women, 6 of whom were in the postmenopausal state. Six subjects in the control group and 3 cases failed to complete the study. Therefore, 18 patients in the intervention group and 15 in the control group completed six months of the follow-up (Fig. 1). The mean age in women and men was 42.11 ± 13.59 years and 51.13 ± 14.33 years, respectively. The mean body mass index (BMI) baseline in the intervention and control groups was 23.72 ± 4.16 kg/m2 and 23.60 ± 4.22 kg/m2, respectively (*P* = 0.938). No significant differences were found between the two groups in baseline characteristics and ESRD etiology, dialysis months, glomerular filtration rate, physical activity, and biochemical characteristics (Table 1).

**Table 1:** Demographic and transplant characteristics of the study population

Parameters	Intervention N (%) Mean ± SD	Placebo N (%) Mean ± SD	*P* value
Female	5 (27.8%)	4 (26.7%)	0.627
Male	13 (72.2%)	11 (73.3%)	
Premenopausal women	2 (11.1%)	2 (13.33%)	0.643
Age (y)	51.22 c 13.53	45.60 ± 15.50	0.275
Female age(y)	48.00 ± 10.24	34.75 ± 14.93	0.157
Male age(y)	52.46 ± 14.785	49.55 ± 14.32	0.630
Time on dialysis(months)^a^	24.00 (22.00)	22.00 (12.5)	0.400
End stage renal disease etiology N (%)			0.828
Hypertensive disease	8 (44.4)	9 (60)	
Diabetic nephropathy	4 (22.2)	3 (20)	
Glomerulonephritis/vasculitis	1 (5.6)	0 (0)	
Cystic/hereditary/congenital diseases	1 (5.6)	0 (0)	
Other	4 (22.2)	3 (20)	
Estimated glomerular filtration rate before transplant (ml/min)	11.265 ± 4.891	10.716 ± 3.992	0.742
Physical activity (total METs)^a^	321.75(618.75)	198 (643.5)	0.919

*METs* Metabolic equivalents

^a^Non-parametric values are reported as median (IQR)

### Change in renal function and bone mineral metabolism

**Fig. 2:**
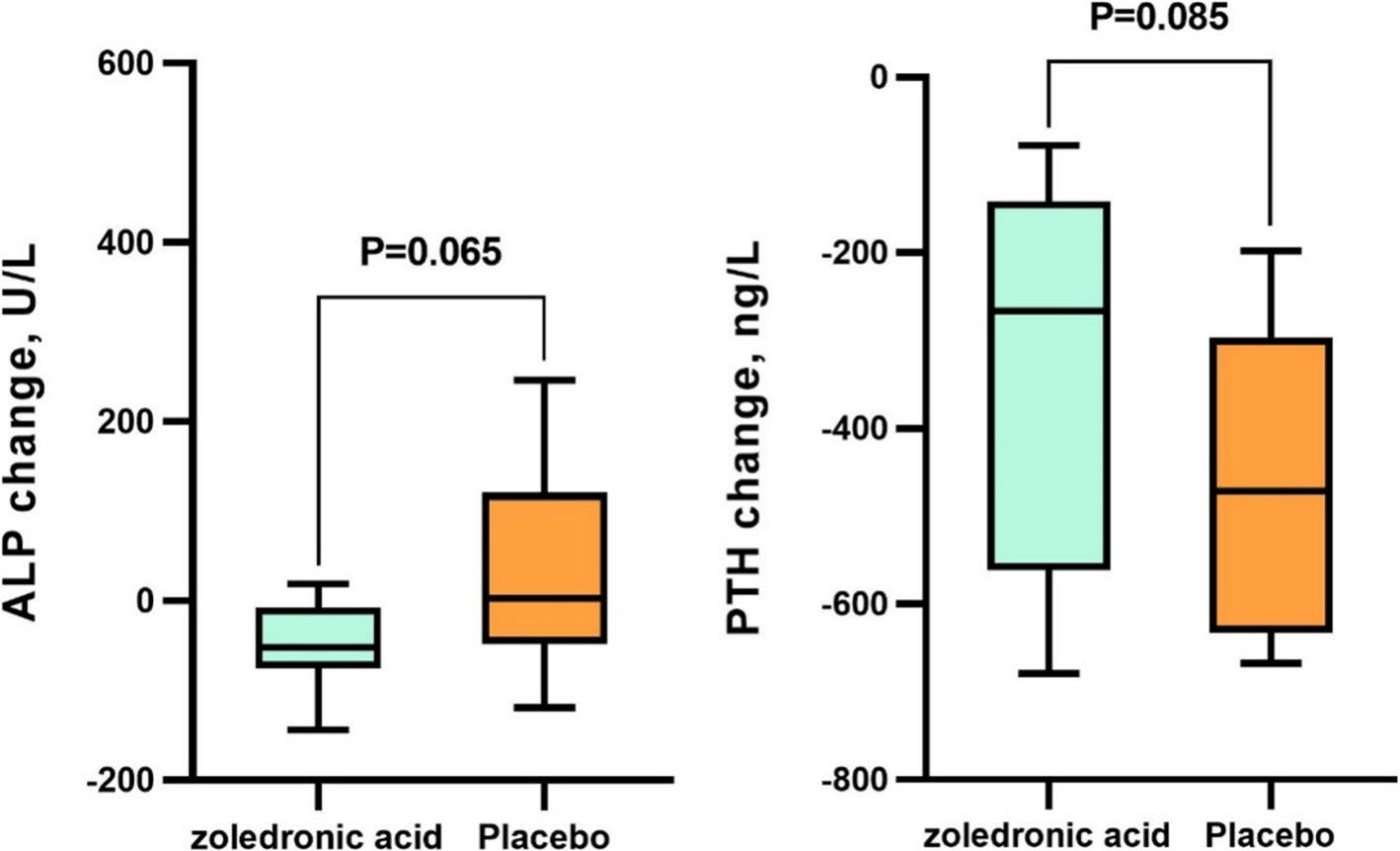
Changes in serum parathyroid hormone and alkaline phosphatase

During the study, there were no significant differences in the mean changes of serum creatinine, blood urea nitrogen, potassium, hemoglobin, phosphorous, PTH, albumin, alkaline phosphates, vitamin D, and estimated glomerular filtration rate (eGFR) between the placebo and zoledronic acid groups (Table 2, Fig. 2). However, the change in serum calcium levels was greater in the placebo group than in the zoledronic acid group (0.50 ± 0.80 vs 0.12 ± 0.62, *p* = 0.025). Furthermore, mean changes in body mass index were not significantly different between the studied groups (Table 2).

**Table 2:** Comparison of body mass index, biochemical characteristics, and estimated glomerular filtration rate between the two groups

parameter	baseline		*P* value	After 6 months		*P* value	Changes		*P* value
	zoledronic acid	placebo		zoledronic acid	placebo		zoledronic acid	placebo	
Body mass index, kg/cm2	23.72 (4.16)	23.60 (4.22)	0.938	25.47 (4.54)	25.98 (4.15)	0.748	1.74 (2.18)	2.38 (2.35)	0.445
Serum creatinine, mg/dL	1.33 (0.26)	1.50 (0.21)	0.083	1.21 (0.15)	1.32 (0.35)	0.352	-0.13 (0.21)	-0.15 (0.32)	0.870
Serum blood urea nitrogen, mg/dL	42.67 (14.49)	38.85 (7.79)	0.396	27.67 (9.51)	30.31 (8.35)	0.429	-15.00 (19.31)	-8.53 (8.39)	0.133
Serum potassium, mg/dL	4.74 (0.91)	4.90 (1.04)	0.648	4.13 (0.63)	4.15 (0.57)	0.947	-0.60 (0.95)	-0.75 (0.87)	0.317
Hemoglobin, mg/dL^a^	11.40 (3.90)	10.80 (3.10)	0.645	10.30 (2.5)	10.00 (4.90)	0.873	-0.85 (2.75)	-1.00 (3.25)	0.968
Serum calcium, mg/dL^a^	8.10 (0.80)	8.00 (1.00)	0.467	8.25 (0.50)	8.40 (0.50)	0.228	0.12 (0.62)	0.50 (0.80)	0.025
Serum phosphorus, mg/dL	4.63 (1.30)	4.18 (0.93)	0.294	2.08 (0.67)	2.16 (0.64)	0.765	-2.55 (1.60)	-2.02 (1.11)	0.317
Serum albumin, mg/dL	3.43 (0.42)	3.34 (0.57)	0.607	3.33 (0.48)	3.34 (0.43)	0.940	-0.10 (0.29)	0.00 (0.371)	0.389
Serum alkaline phosphatase, U/L^a^	207.50 (136.00)	227.00 (111.00)	0.889	182.50 (98.00)	248.00 (231.00)	0.065	-52.00 (67.50)	3.00 (169.00)	0.065
Serum parathyroid hormone, ng/L^a^	311.90 (491.20)	551.90 (392.8)	0.085	45.53 (71.71)	80.57 (57.36)	0.085	-266.36 (419.46)	-471.32 (335.49)	0.085
Serum vitamin D3, ng/mL	23.62 (13.55)	19.73 (11.64)	0.410	55.03 (31.58)	45.97 (27.12)	0.410	31.41 (18.02)	26.24 (15.48)	0.410
estimated glomerular filtration rate, ml/min	59.82 (16.48)	52.25 (9.13)	0.114	65.15 (12.59)	62.64 (19.38)	0.692	5.89 (13.36)	10.92 (15.96)	0.391

^a^Non-parametric values are reported as median (IQR)

### Change in bone mineral density and radiologic evaluation

Using World Health Organization criteria, we found that osteoporosis of the lumbar spine at baseline was present in 27.8% of patients who received zoledronic acid and in 15.4% of those who were administered placebo; also, osteopenia was present in 33.3% and 69.2% of patients, respectively (*P* > 0.05). At the end of the study, the mean value of bone mineral density of intertrochanteric and total femur was found to be significantly higher in the zoledronic group than the placebo group; however, the percentage of changes in bone mineral density in these regions was not significantly different between the two groups (Table 3).

**Table 3:** Comparison of bone mineral density and trabecular bone score between the placebo and zoledronic acid groups

	Baseline			After 6 Months			Changes %		
Parameter	zoledronic acid	placebo	*P* value	zoledronic acid	placebo	*P* value	Zoledronic acid	placebo	*P* value
Radius									
Ultra distal BMD, g/cm2	0.425 (0.062)	0.420 (0.095)	0.850	0.419 (0.052)	0.388 (0.062)	0.140	-0.773 (9.399)	-5.947 (10.831)	0.167
Mid Radius BMD, g/cm2	0.579 (0.058)	0.579 (0.080)	0.973	0.572 (0.054)	0.565 (0.085)	0.791	-1.171 (3.287)	-2.304 (4.556)	0.427
1/3 radius BMD, g/cm2	0.666 (0.072)	0.683 (0.118)	0.630	0.671 (0.067)	0.689 (0.088)	0.522	0.840 (3.438)	2.752 (16.901)	0.642
Total radius BMD, g/cm2	0.557 (0.057)	0.555 (0.074)	0.942	0.550 (0.052)	0.541 (0.077)	0.705	-1.042 (3.967)	-2.500 (3.764)	0.311
Lumbar vertebrae									
Lumbar vertebrae BMD, g/cm2	0.934 (0.143)	0.897 (0.131)	0.466	0.980 (0.156)	0.894 (0.145)	0.128	4.914 (4.590)	-0.235 (7.494)	0.025
Femur									
Femur neck BMD, g/cm2,	0.706 (0.104)	0.638 (0.103)	0.085	0.725 (0.123)	0.650 (0.105)	0.086	2.631 (6.300)	2.088 (6.022)	0.811
Trochanter BMD, g/cm2	0.605 (0.079)	0.558 (0.074)	0.108	0.620 (0.078)	0.561 (0.089)	0.059	2.693 (4.846)	0.293 (8.055)	0.309
Inter trochanter BMD, g/cm2	1.049 (0.134)	0.967 (0.113)	0.086	1.094 (0.133)	0.990 (0.121)	0.035	4.588 (6.470)	2.404 (4.544)	0.305
Total femur BMD, g/cm2	0.856 (0.102)	0.784 (0.091)	0.056	0.891 (0.108)	0.798 (0.101)	0.023	4.166 (4.647)	1.651 (4.701)	0.111
Whole body									
Whole body BMD, g/cm2	1.021 (0.083)	1.019 (0.117)	0.946	1.034 (0.084)	0.998 (0.117)	0.334	1.231 (2.337)	-2.030 (2.999)	0.002
TBS	1.330 (0.124)	1.355 (0.092)	0.541	1.338 (0.121)	1.341 (0.100)	0.940	0.768 (5.213)	-1.02 (3.316)	0.286

*BMD* Bone mineral density, *TBS* Trabecular bone score

**Fig. 3:**
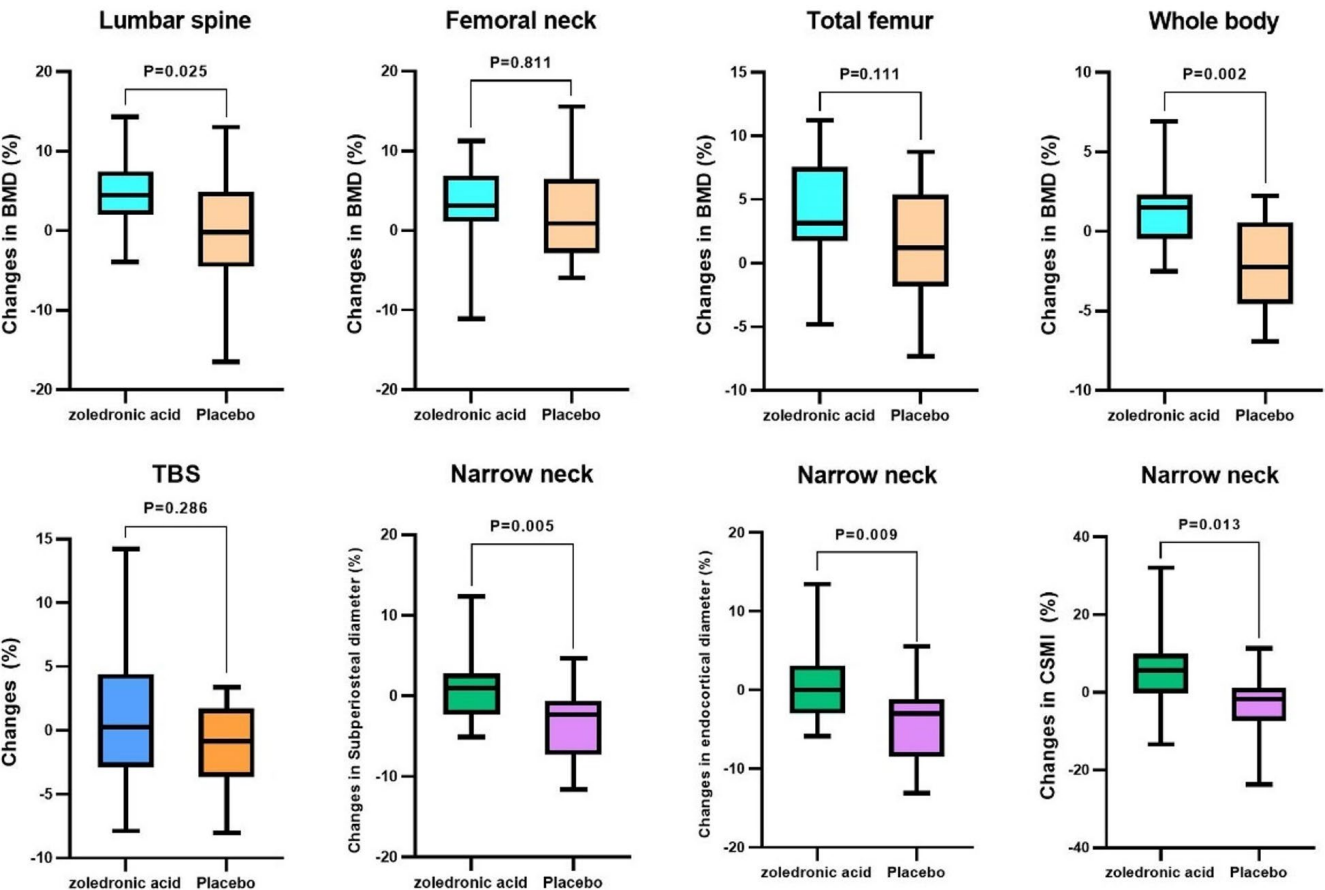
Changes in bone mineral density, trabecular bone score, and hip geometry indices

The mean percentage of changes in the bone mineral density in the lumbar spine was -0.23% and 4.91% in the placebo and zoledronic acid groups, respectively (*P* = 0.02). Also, the mean percentage of changes in bone mineral density in the whole body was -2.03% and 1.23% in the placebo and zoledronic acid groups, respectively (*P* = 0.002) (Table 3, Fig. 3). Seven patients (38.9%) in the zoledronic acid group and 5 (33.3%) in placebo patients showed a vertebral fracture at baseline, with no differences between the groups (*P* = 0.741). Six months after kidney transplantation, 16.7% (3) of the patients in the zoledronic acid group and 20% (3) in the placebo group developed vertebral fractures (*P* = 0.577).

### Change in the trabecular bone score and hip geometry indices

At baseline, trabecular bone score and hip geometric analysis indices were not significantly different between the zoledronic acid and placebo groups (Tables 3 & 4). Our study could not detect a significant difference in TBS changes during the six months of treatment with zoledronic acid combined with calcium and vitamin D3 compared with the patients who received only calcium and vitamin D3 after kidney transplant (Table 3, Fig. 3). A significant difference was found in the percentage of changes in subperiosteal diameter, endocortical diameter, and CSMI in the narrow neck between the two groups from the beginning to 6 months, with positive changes in the zoledronic acid group and negative changes in the control group (Table 4, Fig. 3).

**Table 4:** Baseline hip geometric measurements and changes at 6 months in the placebo and zoledronic acid groups

	Baseline			After 6 Months			Changes %		
Parameter	zoledronic acid	placebo	*P* value	zoledronic acid	placebo	*P* value	zoledronic acid	placebo	*P* value
NN Subperiosteal diameter (cm) Width	3.527 (0.376)	3.693 (0.375)	0.233	3.578 (0.436)	3.557 (0.350)	0.889	1.409 (4.519)	-3.580 (4.387)	0.005
IT Subperiosteal diameter (cm) Width	5.608 (0.524)	5.769 (0.540)	0.412	5.614 (0.483)	5.736 (0.564)	0.522	0.248 (3.736)	-0.444 (5.594)	0.682
FS Subperiosteal diameter (cm) Width	3.211 (0.304)	3.166 (0.250)	0.672	3.211 (0.288)	3.213 (0.303)	0.990	0.093 (2.642)	1.420 (4.158)	0.286
NN endocortical diameter (cm)	3.201 (0.396)	3.402 (0.400)	0.175	3.237 (0.474)	3.252 (0.367)	0.925	1.0287 (5.329)	-4.260 (5.019)	0.009
IT endocortical diameter (cm)	4.881 (0.525)	5.084 (0.583)	0.318	4.868 (0.481)	5.066 (0.612)	0.320	-0.049 (4.570)	-0.241 (5.832)	0.919
FS endocortical diameter (cm)	2.128 (0.406)	2.171 (0.341)	0.760	2.073 (0.395)	2.200 (0.425)	0.400	-2.346 (7.611)	1.247 (8.723)	0.232
NN Cross-sectional area (cm2)	2.853 (0.487)	2.669 (0.358)	0.258	2.980 (0.519)	2.706 (0.377)	0.117	4.528 (5.880)	1.382 (4.937)	0.128
IT Cross-sectional area (cm2)	4.882 (0.800)	4.562 (0.681)	0.253	5.011 (0.821)	4.528 (0.663)	0.092	2.860 (6.173)	-0.298 (11.076)	0.319
FS Cross-sectional area (cm2)	4.483 (0.755)	4.130 (0.710)	0.199	4.667 (0.789)	4.237 (0.636)	0.117	4.385 (7.572)	3.118 (6.477)	0.630
NN CSMI (cm4)	2.928 (0.921)	2.812 (0.637)	0.699	3.079 (0.980)	2.741 (0.715)	0.300	5.651 (9.761)	-3.184 (8.222)	0.013
IT CSMI(cm4)	14.031 (4.450)	13.435 (3.900)	0.702	14.626 (4.655)	13.158 (3.947)	0.364	5.238 (13.763)	-0.202 (20.450)	0.382
FS CSMI(cm4)	4.363 (1.515)	3.875 (1.057)	0.327	4.415 (1.458)	3.953 (0.923)	0.324	1.821 (7.773)	3.298 (12.345)	0.686
NN Section modulus (cm3)	1.507 (0.385)	1.379 (0.221)	0.253	1.583 (0.408)	1.398 (0.273)	0.166	5.254 (7.567)	0.965 (8.197)	0.143
IT Section modulus (cm3)	4.197 (1.024)	3.969 (0.956)	0.535	4.436 (1.198)	3.952 (0.983)	0.243	5.840 (11.981)	0.401 (16.711)	0.299
FS Section modulus (cm3)	2.583 (0.649)	2.343 (0.486)	0.270	2.643 (0.636)	2.366 (0.433)	0.186	2.647 (5.850)	1.972 (11.894)	0.852
NN Cortical thickness (cm)	0.163 (0.027)	0.144 (0.023)	0.052	0.168 (0.033)	0.153 (0.023)	0.152	2.730 (8.149)	6.234 (5.888)	0.198
IT Cortical thickness (cm)	0.363 (0.054)	0.343 (0.050)	0.301	0.373 (0.058)	0.336 (0.058)	0.098	2.940 (6.903)	-1.666 (10.845)	0.159
FS Cortical thickness (cm)	0.540 (0.103)	0.499 (0.093)	0.286	0.568 (0112)	0.504 (0.092)	0.105	5.709 (10.434)	1.433 (8.738)	0.239
NN Buckling ratio	12.172 (3.071)	14.492 (3.385)	0.056	12.055 (4.118)	13.023 (2.462)	0.457	-2.088 (10.148)	-8.711 (11.500)	0.101
IT Buckling ratio	9.294 (1.749)	10.062 (1.968)	0.262	8.938 (1.670)	10.284 (2.690)	0.096	-3.502 (6.768)	1.595 (10.392)	0.108
FS Buckling ratio	3.189 (0.741)	3.423 (0.876)	0.428	3.000 (0.637)	3.469 (1.120)	0.149	-5.080 (11.120)	0.896 (12.879)	0.177
Neck Shaft Angle	126.06 (6.629)	127.08 (6.487)	0.672	124.444 (5.982)	125.538 (5.636)	0.611	-1.158 (4.397)	-1.128 (3.393)	0.983

*NN* Narrow neck, *IT* Inter-trochanteric, *FS* Femoral shaft, *CSMI* Cross-sectional moment of inertia

## Discussion

Bone strength can be described as the ability to resist fractures, which is based on bone quality as well as bone mass. The quality of the bone is affected by bone microarchitecture, geometry, and tissue material properties [24]. The Dual-energy X-ray absorptiometry method is commonly used in clinical practice for measuring bone mass. DXA converts the three-dimensional bone structure into a two-dimensional image from which BMD measurements are derived. With these explanations, this approach fails to consider crucial structural parameters inclusive of bone geometry and internal architecture, and it has been demonstrated to possess inadequate sensitivity when predicting the fracture [25]. Consequently, conducting a comparative analysis of bone mineral density, trabecular bone score, and femoral structural analysis indices prior to and subsequent to drug administration, facilitates a more comprehensive investigation of the drug's preventive mechanism against bone fractures.

Based on the findings of this study, the percentage of change in the spine and whole body BMD was significantly different between the patients who received zoledronic acid and those who were administered placebo; in patients who received placebo, these changes were negative, whereas those who received zoledronic acid showed positive changes. However, no significant difference was seen in the percentage of changes in the femur as a cortical bone [26] between the two groups. This finding is consistent with that of Haas et al. [15], who showed a beneficial effect of zoledronic acid in improving the calcium content of cancellous bone after kidney transplantation. This is also supported by previous studies [27, 28] that showed the role of third-generation bisphosphonates in increasing cancellous bone formation that could be only measured by DXA image of the cancellous vertebra rather than DXA results of the cortical femur neck. Therefore, given that the stability and performance of the bone depend more on trabecular mineralization and architecture than on cortical mineralization [29], zoledronic acid might be of benefit to kidney transplant patients for improving bone strength.

TBS is a textural index from spine DXA images that predict fractures independent of areal bone mineral density (BMD). A few studies have been conducted on how TBS is related to trabecular microarchitecture in patients with kidney transplant. In this study, no significant difference in TBS change was seen between the two groups. This result is in the same line with those of previous studies, which found that TBS did not provide clinically useful information regarding the effects of bisphosphonate on skeletal health [30], as Popp et al. did not find a TBS increase beyond the least significant change despite BMD increases in the lumbar spine in patients who received zoledronic acid [31]. This is not unexpected because one would assume a greater enhancement in BMD, particularly with antiresorptive therapy, due to increased mineralization and filling of the remodeling space than improvement in the trabecular microstructure as assessed by TBS [32]. Therefore, although the lumbar spine TBS might be a valuable tool for assessing bone quality and fracture risk prediction in kidney transplant recipients [8], it cannot be used to monitor the skeletal effects of zoledronic acid.

To the best of our knowledge, this is the first study to examine the impact of zoledronic acid on the bone geometric properties of the hip in kidney transplant patients. The present study showed a significant difference in the percentage of changes of subperiosteal diameter, endocortical diameter, and CSMI at the narrow neck between the cases and controls, and zoledronic acid appeared to enhance these parameters in comparison with placebo. This is in line with previous studies that showed the association of bisphosphonate with an improvement of geometry in the proximal femur [33–37]; although there is a difference in the type of improved parameters in these studies, and ours, this improvement was seen only in the subperiosteal diameter, endocortical diameter, and CSMI at the narrow neck. This could be explained by different methods, such as different types of bisphosphonates, duration of treatment, population study, underlying diseases, and imaging modalities. Based on the results of our study, despite the lack of improvement in the femoral bone mineral density, HSA showed a positive effect on the geometric parameters of the bone strength in the narrow neck in post-renal transplant patients taking zoledronic acid, so HSA might be a useful tool to predict the effect of zoledronic acid on the bone strength of kidney transplant recipients, as although we could not detect any protective effect of zoledronic acid on bone loss in femoral sites through DXA measurements, we were able to detect beneficial effects in more specific sub-sections of the proximal femur through the use of HSA.

Supposing intact coupling, we anticipated that the zoledronic acid group would show decreased bone formation parameters as determined by alkaline phosphatase measurements and bone resorption [38]. Also, normal bone formation and normal-to-increased bone resorption were expected for the control group. We confirmed the decrease in alkaline phosphatase in the zoledronic acid group; however, we could not demonstrate a significant difference in alkaline phosphatase change between the two groups. This result agrees with previous studies that showed no significant difference in bone-specific alkaline phosphatase changes in the zoledronic acid and placebo groups [15, 39]. It is important to take into account age, gender, race, incidental fractures, and circadian rhythms when assessing these parameters [40]. Also, the basic calcium and vitamin D supplements of both groups could have played a role in achieving this result [41].

Because this intervention was implemented for both sexes, a wide range of ages, and different causes of ESRD, the findings can be attributed to men and women with a wide range of ages and underlying causes of renal failure leading to kidney transplantation; However, due to the relatively small sample size, the variability of the study population might affect the ability to detect a treatment effect and be considered as a potential limitation. An additional strength of this study, was that All participants were recruited at the same early time point after kidney transplantation. Additionally, it is important to note that with the advancements in Immunosuppression in kidney transplantation, there may be less bone loss expected [42]. Hence, it is necessary to conduct additional research with larger sample sizes. Finally, to evaluate the bone strength using hip structural analysis, we need to consider some limitations, including difficulties in accurately positioning the femur and locating precise edge margins of blurred and noisy DXA scan images in addition to the lack of DXA device design due to its 2-dimensional nature to assess hip geometry, that could lead to misinterpretation of HSA values. Also, it’s important to note that while the majority of BMD decline happens within the first six months following kidney transplantation, which puts individuals at a higher risk for fractures, there is a continued decrease in BMD between six and twelve months after the procedure [43, 44]. Hence, a longer duration of follow-up in kidney transplant recipients on bisphosphonate therapy may be beneficial for assessing potential improvements in BMD and hip geometric parameters; therefore, further studies with longer follow-up and methodological considerations are suggested.

## Conclusion

In conclusion, our results revealed that a single administration of zoledronic acid may ameliorate the bone loss at the lumbar spine and whole body and maintain the subperiosteal diameter, endocortical diameter, and CSMI as parameters of bone strength at the narrow neck in post-transplant patients during 6 months of follow-up. However, Further research is required, particularly to assess the long-term impact of bisphosphonates in preventing fractures, before their prophylactic usage after kidney transplantation can be widely recommended.

## Data Availability

The datasets used and/or analyzed during the current study are available from the corresponding author on reasonable request.

## Abbreviations

BMD: Bone mineral density
TBS: Trabecular bone score
CSMI: Cross-sectional moment of inertia
ESRD: End stage renal disease
HSA: Hip structural analysis
DXA: Dual-energy X-ray absorptiometry
BMI: Body mass index
PTH: Parathyroid hormone
ROI: Region of interest
NN: Narrow neck
IT: Intertrochanter
FS: Femoral shaft
CSA: Cross-sectional area
BR: Buckling ratio
eGFR: Estimated glomerular filtration rate
